# Compressive hyperspectral microscopy for cancer detection

**DOI:** 10.1117/1.JBO.28.9.096502

**Published:** 2023-09-09

**Authors:** Yaniv Oiknine, Marwan Abuleil, Eugene Brozgol, Isaac Y. August, Iris Barshack, Ibrahim Abdulhalim, Yuval Garini, Adrian Stern

**Affiliations:** a Ben-Gurion University of the Negev, School of Electrical and Computer Engineering, Electro-Optics and Photonics Engineering Department, Beer-Sheva, Israel; b Bar-Ilan University, Physics Department, Faculty of Exact Sciences, Ramat Gan, Israel; c Shamoon College of Engineering, Department of Electrical Engineering and Physics, Beer Sheva, Israel; d Tel-Aviv University, Sackler Faculty of Medicine, Tel-Aviv, Israel; e Sheba Medical Center, Department of Pathology, Ramat Gan, Israel; f Technion IIT, Biomedical Engineering Faculty, Haifa, Israel

**Keywords:** hyperspectral imaging, microscopy, cancer detection, liquid crystal modulators, compressive sensing, digital pathology

## Abstract

**Significance:**

Hyperspectral microscopy grants the ability to characterize unique properties of tissues based on their spectral fingerprint. The ability to label and measure multiple molecular probes simultaneously provides pathologists and oncologists with a powerful tool to enhance accurate diagnostic and prognostic decisions. As the pathological workload grows, having an objective tool that provides companion diagnostics is of immense importance. Therefore, fast whole-slide spectral imaging systems are of immense importance for automated cancer prognostics that meet current and future needs.

**Aim:**

We aim to develop a fast and accurate hyperspectral microscopy system that can be easily integrated with existing microscopes and provide flexibility for optimizing measurement time versus spectral resolution.

**Approach:**

The method employs compressive sensing (CS) and a spectrally encoded illumination device integrated into the illumination path of a standard microscope. The spectral encoding is obtained using a compact liquid crystal cell that is operated in a fast mode. It provides time-efficient measurements of the spectral information, is modular and versatile, and can also be used for other applications that require rapid acquisition of hyperspectral images.

**Results:**

We demonstrated the acquisition of breast cancer biopsies hyperspectral data of the whole camera area within ∼1  s. This means that a typical $1\times 1\;{\mathrm{cm}}^{2}$ biopsy can be measured in ∼10  min. The hyperspectral images with 250 spectral bands are reconstructed from 47 spectrally encoded images in the spectral range of 450 to 700 nm.

**Conclusions:**

CS hyperspectral microscopy was successfully demonstrated on a common lab microscope for measuring biopsies stained with the most common stains, such as hematoxylin and eosin. The high spectral resolution demonstrated here in a rather short time indicates the ability to use it further for coping with the highly demanding needs of pathological diagnostics, both for cancer diagnostics and prognostics.

## 1 Introduction

### 1.1 Cancer Digital Diagnostics by Spectral Imaging

Digital pathology has evolved during the last decade to meet the growing demand for cancer diagnostics. The standard pathological analysis is performed by eye-balling through a brightfield or fluorescence optical microscope. Whole slide imaging systems that scan biopsies were developed that provide pathologists with high-quality images that can be diagnosed on the computer screen. These systems are based on measuring three intensities for each pixel of the image, which includes information in the red, green, and blue (RGB) spectral channels.^1^ Although multiple publications demonstrate high-quality images, its computer-based analysis is still not in use for medical purposes.^2^^,^^3^

One direction that can address this deficiency is to increase the amount of information gathered from the biopsies by measuring spectral images that provide the light spectrum at each pixel of biopsies.^4^^,^^5^ Measuring spectral information from cancer biopsies was tested before by different methods, but most of the works concentrated on measurements at the tissue level and not on the sub-cellular features.^6^^,^^7^ Furthermore, most spectral imaging systems have a long acquisition time that limits their usage for biopsies that require capturing very large images (e.g., on the order of 40,000×40,000  pixels and more). Lately, a new method for spectral imaging has been introduced and demonstrated its applicability for cancer detection.^8^ Nevertheless, there is still a need for further improvements to confront the broad range of pathological stains, the variability of samples, and their complexity.

### 1.2 Spectral Microscopy

Measuring spectral images is not trivial, as the final data are three-dimensional, namely I(x,y,λ), while cameras, such as CCD or CMOS, only provide array detection (i.e., two-dimensional). Accordingly, different methods were developed that vary from the measurement of only a few spectral bands in the required spectral range to the measurements of high spectral resolution by measuring one line of the image at a time, a procedure that takes a very long time. The conventional methods for spectral imaging use a series of narrowband filters that are switched one at a time during the measurement of each field of view, a procedure that takes much time. There are also electronically variable filters, such as liquid crystal tunable filter (LCTF) or acousto-optic tunable filter, that still necessitate switching the filter through many spectral bands for every field of view. Although these filters can be fast enough, the acoustooptic one is bulky and requires power of at least 1 W, while the LCTF usually has lower light throughput. Biological applications of LCTFs and other liquid crystal (LC) devices for skin cancer and pathology applications were discussed before.^9^^–^^12^ Another set of methods is based on Fourier spectroscopy,^13^ which provides a rather rapid measurement, and there are even methods for one-shot spectral imaging, although of a rather small region of interest from the sample.^14^ For a comprehensive discussion on different methods for spectral imaging, see Ref. 4.

### 1.3 Compressive Sensing

The design of our system and the reconstruction algorithms follow the compressive sensing (CS) framework.^15^^–^^18^ The CS theory prescribes a framework to capture and recover sparse signals from fewer measurements than required by traditional systems that have been designed to comply with the Shannon–Nyquist sampling theorem. In our context, we design a spectral sensing process described mathematically as

g=Φf, (1)

where $f\in R^{N}$ denotes the spectral vector to be measured, $\Phi \in R^{M\times N}$ is the sensing matrix modeling the sensing operator, and $g\in R^{M}$ is the vector of the spectrally multiplexed measured signal, which is compressed, i.e., M<N. The inversion of Eq. (1) is possible, i.e., the spectrum can be numerically reconstructed from fewer measurements if the spectrum has a sparse representation, meaning, f=Ψα, where α is a K-sparse vector (that is, contains K≪N non-zero elements) and Ψ is the sparsifying operator. The sparsifying operator is in general a mathematical basis (discrete cosine transform, wavelet, curvelets, etc.) or a learned dictionary.^19^ In order to solve Eq. (1), that is, to reconstruct the original spectral signal f from the measurement vector g, numerous CS algorithms are available.^20^

The recovery process requires specifying the sensing operator Φ that maps the original spectral signal onto the measured spectrally multiplexed signal. In our system, the sensing matrix is physically realized by utilizing an LC variable retarder, which is specified by measuring its spectral transmission between two polarizers for different voltages (see Sec. 2.2.).

## 2 Compressive Spectral Imaging Microscope Design

### 2.1 CS Spectral Microscope Design

In the last few years, we presented several compressive hyperspectral (HS) systems^21^^–^^25^ that use various modified spectral modulators designed to capture compressed spectral data. One such system is the compressive sensing miniature ultraspectral imaging (CS-MUSI) system,^21^^–^^24^ which performs spectral multiplexing of the captured image by applying various voltages on a thick LC cell (LCC). Each voltage determines one row in the sensing matrix Φ in Eq. (1). The CS-MUSI was designed for remote sensing applications and has been demonstrated to capture ultraspectral images with an optical compression ratio of up to 40:1. The compressive spectral imaging microscopy presented in the current paper uses a working principle similar to that of the CS-MUSI, but with an essentially different system design; instead of spectrally encoding the light propagating from the object to the sensor, we spectrally encode the illumination of the sample. By this, we realize the same CS sensing model described in Eq. (1) but retain the well-designed imaging optics of the microscope. Another improvement of the system presented here is the LC design and its voltage control, which allowed us to reduce the acquisition time of M compressive measurements to <1  s (Sec. 2.2).

**Fig. 1:**
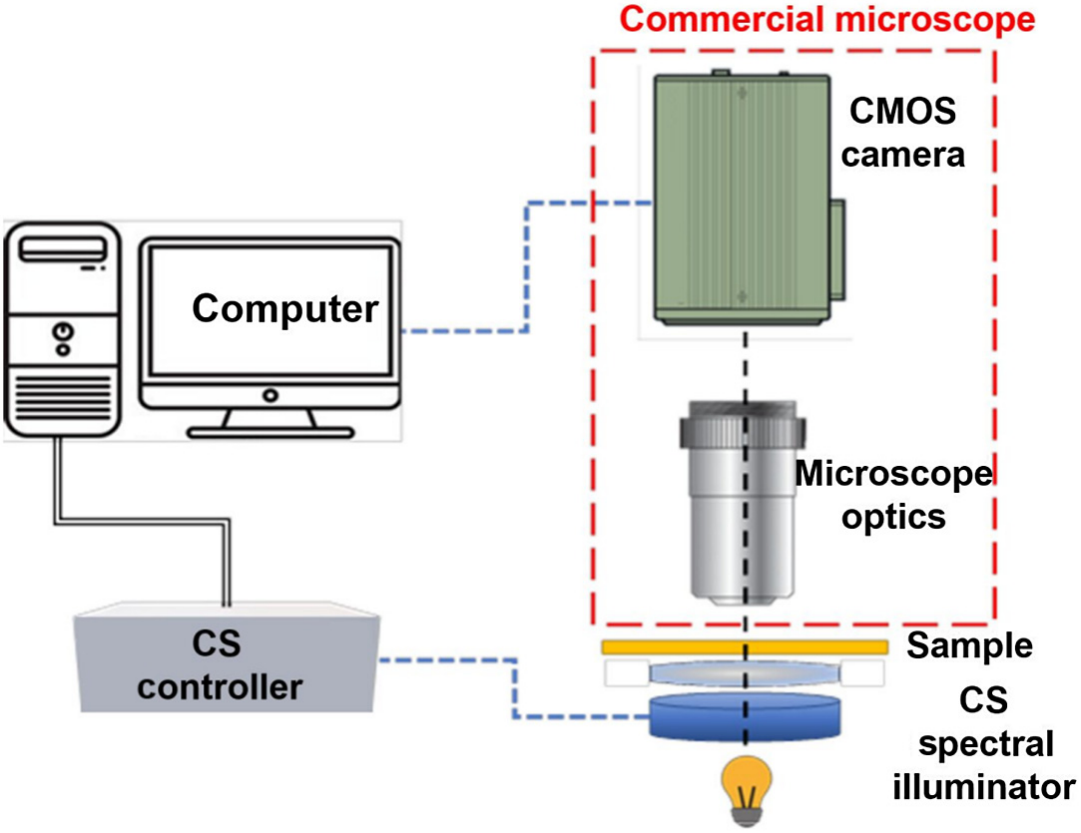
COHSM schematic drawing. The sample is illuminated sequentially by M spectrally modulated patterns. For each spectrally encoded illumination pattern, the grayscale image is collected with a conventional microscope.

Figure 1 depicts a schematic drawing of our system compressive hyperspectral microscopy (COHSM) system. The sample is illuminated sequentially by spectrally modulated patterns generated by a specially designed LCC. The LCC design is described in Sec. 2.2. A total of M spectrally modulated images are taken following the CS framework (Sec. 1.3). As a result, the spectrum at each pixel in the image is compressively sensed. Then the whole spectrum containing N spectral bands is reconstructed by applying a CS reconstruction algorithm for each pixel.

**Fig. 2:**
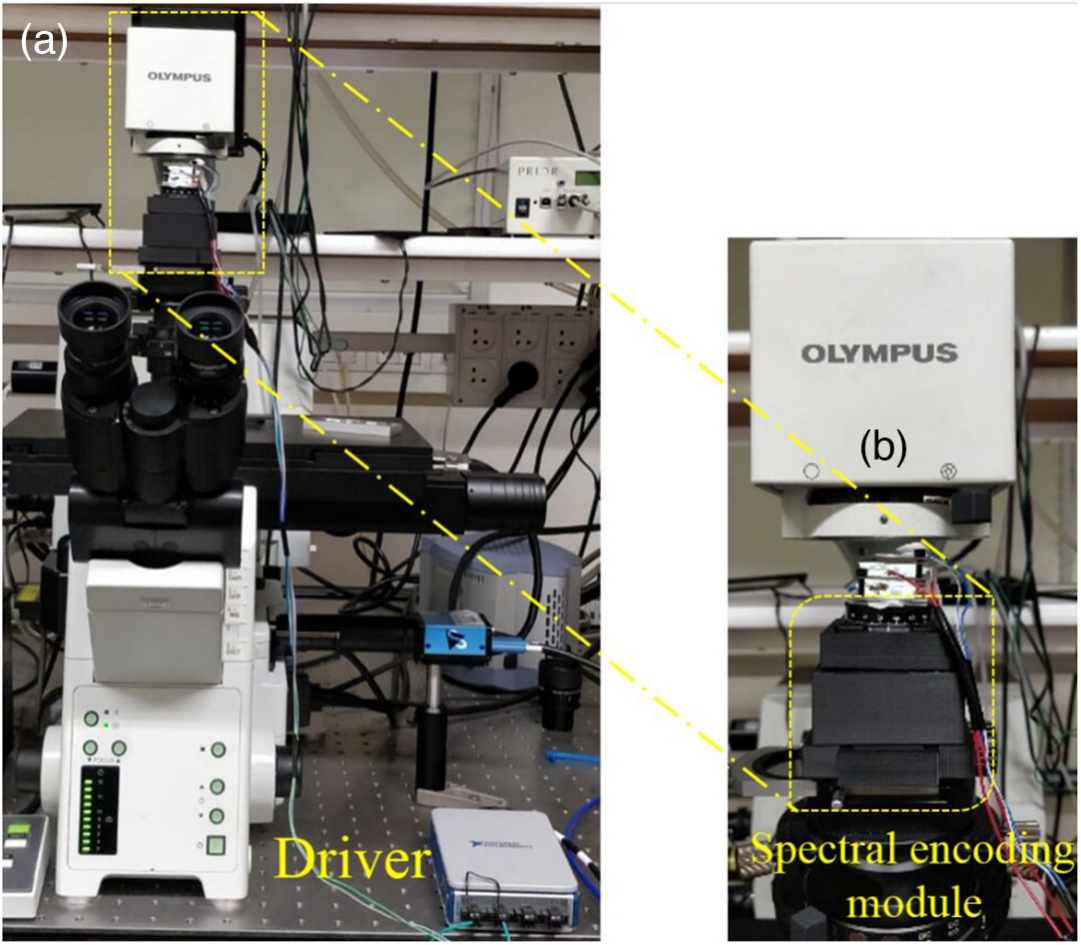
The spectral imaging system. It is based on a commercial Olympus IX81 microscope (a) where we plugged in a SM into the illumination arm (b).

We have demonstrated the COHSM principle on an Olympus IX81 inverted microscope [Fig. 2(a)]. The main hardware components of our method consist of our special illumination unit [Fig. 2(b)] and its driver. A laptop computer is used for the acquisition control and the image reconstruction. The illumination unit and the microscope camera are controlled by a LabView program code. The reconstruction of the spectral images is performed by a CS algorithm realized in MATLAB.

Notice that the only hardware change in the Olympus microscope is the extra unit in the illumination arm; therefore, we preserve the high spatial imaging capabilities of the commercial microscope. This property is verified and confirmed experimentally in Sec. 3.1.

The illumination module generates a set of M spectrally encoded patterns. The complete set of patterns is generated within <1  s owing to the unique LC design and its operation described in Sec. 2.2. Thus the acquisition of the compressed spectral image can be made at a rate of one spectral frame per second. From the captured data, we have been able to reconstruct spectral images with 710 spectral bands with full spatial resolution. In our experiments, we limited the spectral range to 400 to 750 nm.

### 2.2 LC Design

**Fig. 3:**
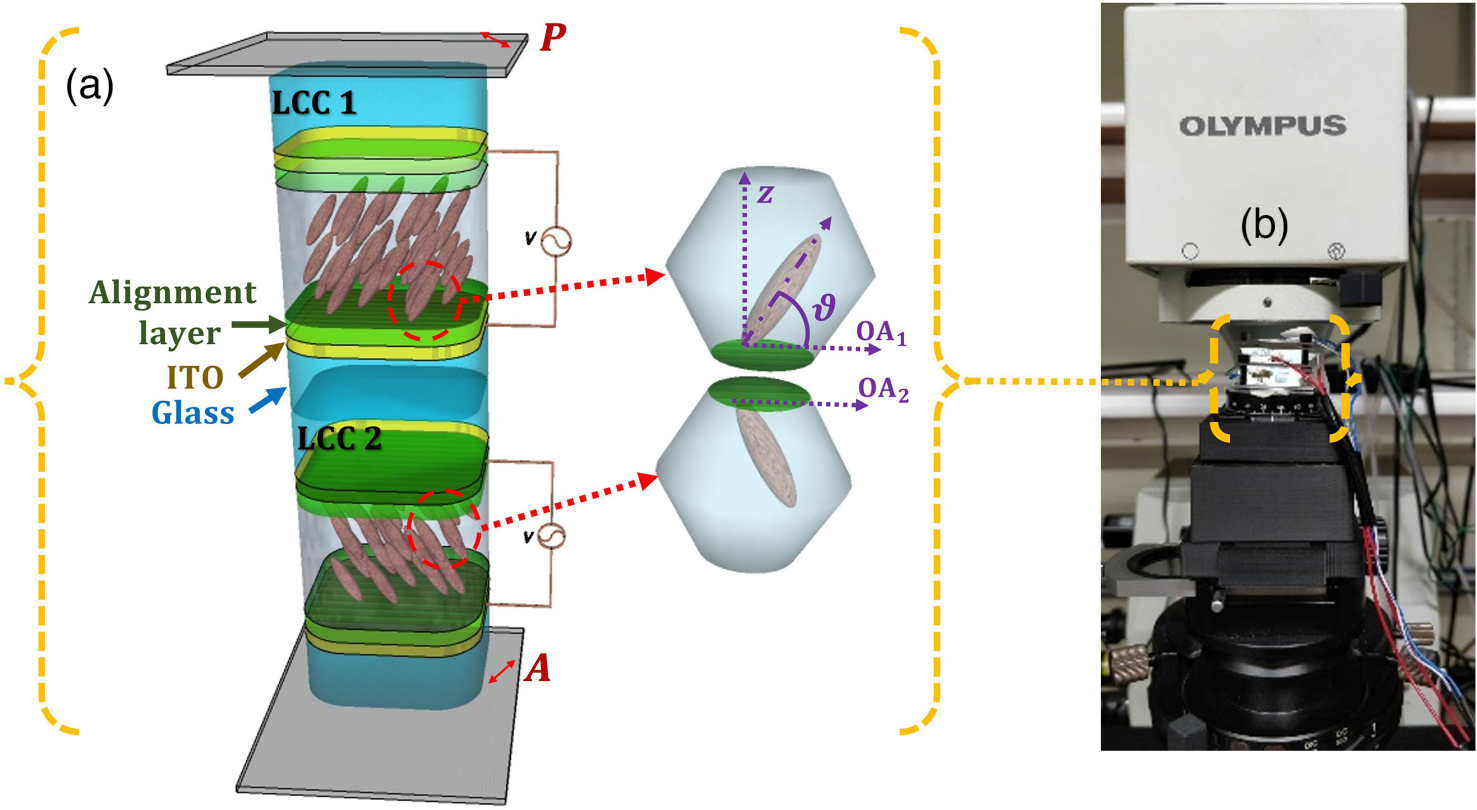
(a) Schematic structure of the SM composed of two anti-parallel aligned LCCs (one is a mirror image of the other) between crossed polarizers. P and A represent the crossed polarizer and analyzer, whereas ${\mathrm{OA}}_{1}$, ${\mathrm{OA}}_{2}$ represent the LCCs optical axis that is oriented 45 deg to the polarizer. (b) The SM is fixed on the condenser of a commercial Olympus BX53 microscope using a mount M.

Our spectral encoding module includes two anti-parallel aligned E7 LCCs with 50 and 25  μm LC thicknesses, instead of using a single LCC with 75  μm thickness. Replacing a thick LCC with two thinner LCCs reduces the response time by a factor of 2.2. Also placing the two cells in a configuration such as one is a mirror image of the other improves the cell viewing angle.^26^^,^^27^ Figure 3(a) shows the schematic structure of the anti-parallel aligned LCC. By applying an electrical field along the z axis, the tilt angle (ϑ) profile of the LC molecules changes.^28^ As a result, the LC birefringence (Δn) profile also changes, which provides the LCC total retardation tunability. When the LCCs are oriented at 45 deg between crossed polarizers, the transmission of the spectral encoding module along the z axis is

$$T=\sin^{2}(\Gamma_{\text{total}}/2),$$ (2)

where $\Gamma_{\text{total}}$ represents the total retardation of the stock, which equals the summation of the retardation of the two LCCs. Higher applied voltages lead to larger molecule’s tilt angle and lower birefringence and hence retardation. As a result, the number of fringes in the spectral modulation (SM) reduces also. Figure 3(b) shows the SM assembly positioned on the condenser of a commercial Olympus BX53 microscope using a specially designed mount and prepared using a 3D printer.

**Fig. 4:**
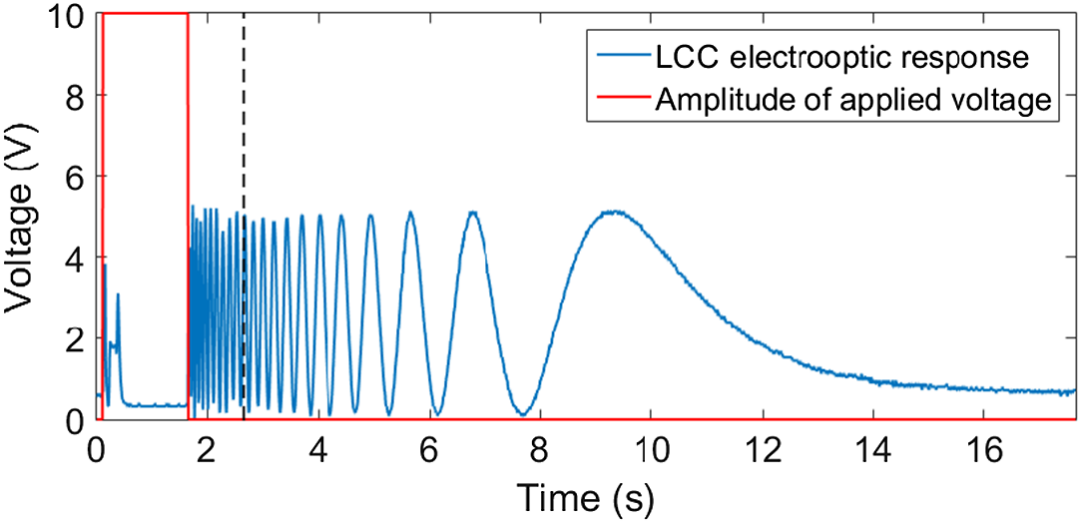
The electrooptic response of the SM at 635 nm (blue) in respons to applied 1 kHz AC square wave voltage. Red represents the amplitude of the applied voltage. The black line represents the 1 s window after switching off the voltage during which all the image acquisitions are accomplished.

**Fig. 5:**
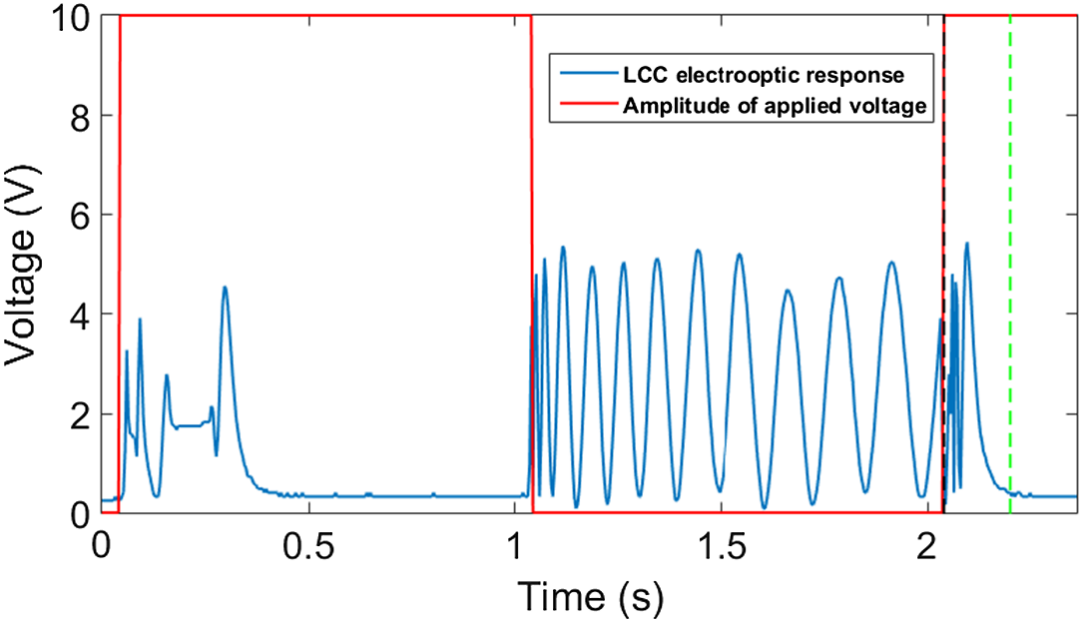
The electrooptic response of the SM at 635 nm (blue) in response to applied 1 kHz AC square wave voltage. The red line represents the amplitude of the applied voltage, the black line represents the 1 s window after switching off the voltage during which all the image acquisitions are accomplished. The green line represents the point where the electrooptic response gets stabilized.

A square wave AC voltage is applied on both LCCs between 0 and 10 V to operate the spectral encoding module. The electrooptic response of the spectral encoding module has been tested at 635 nm while the results appear in Figs. 4 and 5. As the first step, the applied voltage turns on from 0 to 10 V, as Fig. 4 shows, which leads to maximum LC molecules reorientation or reaches the low number of fringes in the SMs in ∼310  ms rise time. The SMs were captured during the first 1 s window after turning off the voltage. Although the decay time is >1  s, the modulation change after 1 s is small and slower. As a result, it is sufficient to capture the modulation in equal intervals during the first 1 s. At each such interval, the voltage falling on the LC is different. For our purposes of the computational algorithm, there is no need to know the exact absolute value of the voltage falling on the LC. This methodology was first used by Hegyi and Martini^26^ to perform Fourier transform spectroscopy using LCC. By replacing the 25  μm LCC with a thicker one, more SMs can be achieved in the 1 s window without affecting the system speed. If we turn on the voltage to 10 V immediately after the 1 s window, the device’s electrooptic response stabilizes after ∼160  ms, as Fig. 5 shows, leading to faster measurement. In other words, multiple scans or video capture can be achieved with 1.16 s per frame.

**Fig. 6:**
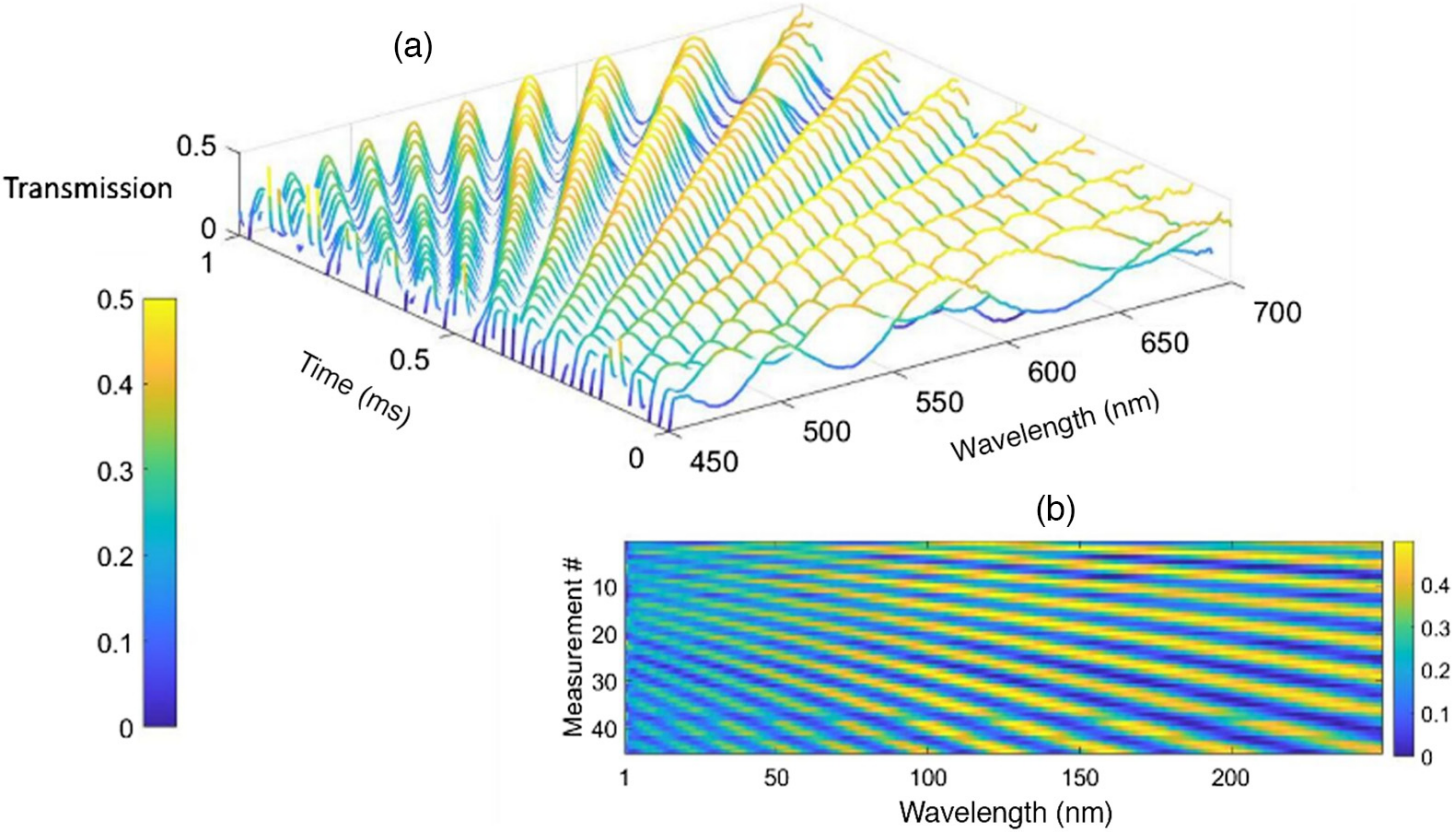
(a) Measured transmission map and (b) map of the sensing matrix.

The measured transmission map and sensing matrix Φ of the system are presented in Fig. 6. The transmission map presents all the transmission vector that we measured in the calibration step during 1 s window.

## 3 Optical Performances

As already mentioned, our spectral imaging method is designed to provide a spatial image quality similar to that of the commercial microscope employed. This owes to the fact that we do not change the well-designed imaging objective of the commercial microscope, while only applying a spatially uniform, spectrally encoded illumination. Owing to the CS design, the spectral resolution is on the nanometer scale, and owing to the multiplexed measurements, the optical throughput is two orders of magnitude larger than that of band-selective-based spectrometers. In this section, we verify quantitatively and explain these properties.

### 3.1 Spatial Resolution

**Fig. 7:**
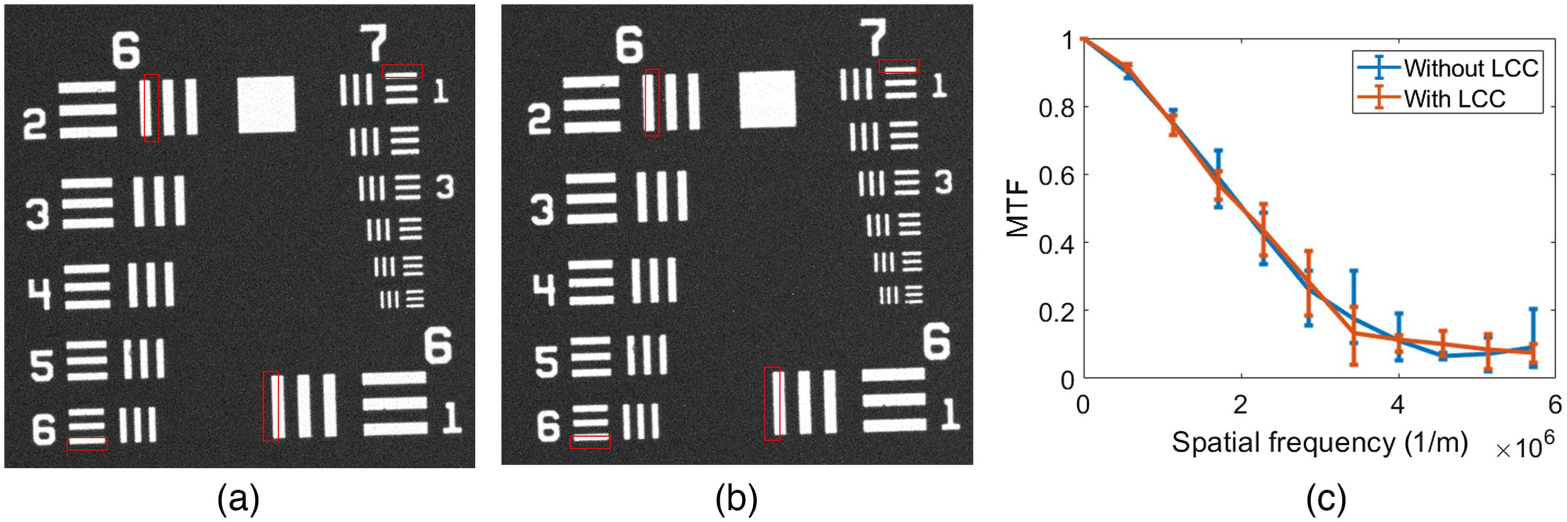
(a) Spatial resolution of COHSM. USAF resolution test target image taken (a) without and (b) with the SM module placed in the illumination arm of a commercial Olympus IX81 microscope. (c) Comparison of the MTFs from (a) and (b). It can be seen that the inclusion of the SM does not deteriorate the MTF of the microscope.

To evaluate the spatial performance of our method, we took an image of a resolution test target (Thorlabs Negative 1951 USAF Resolution Test Target) with our system. We then compared it with that obtained by the native microscope (i.e., without the LCC in the illumination arm). As shown in Fig. 7, the two images look similar, and there is no observable difference between the images. Both images’ resolution is similar, as demonstrated by the modulation transfer function (MTF) plots shown in Fig. 7(c). The MTFs were calculated using the edge response method evaluated on various regions in the field of view. Briefly, with this method, the average edge spread function (ESF) is evaluated from the edge of the bars marked in red. The derivative of the ESF yields the line spread function, from which a Fourier transform obtains the optical transfer function (OTF). Finally, the absolute value of the OTF gives the MTF. Figure 7(c) presents a comparison of the MTF plots for both cases, without and with LCC, and different edges in the taken image (marked by red rectangles). It can be seen that the average MTF of the system with the LCC included in the illumination arm is almost identical to that of the native microscope. Also no changes in the spatial resolution over the image field can be noticed.

### 3.2 Spectral Resolution

**Fig. 8:**
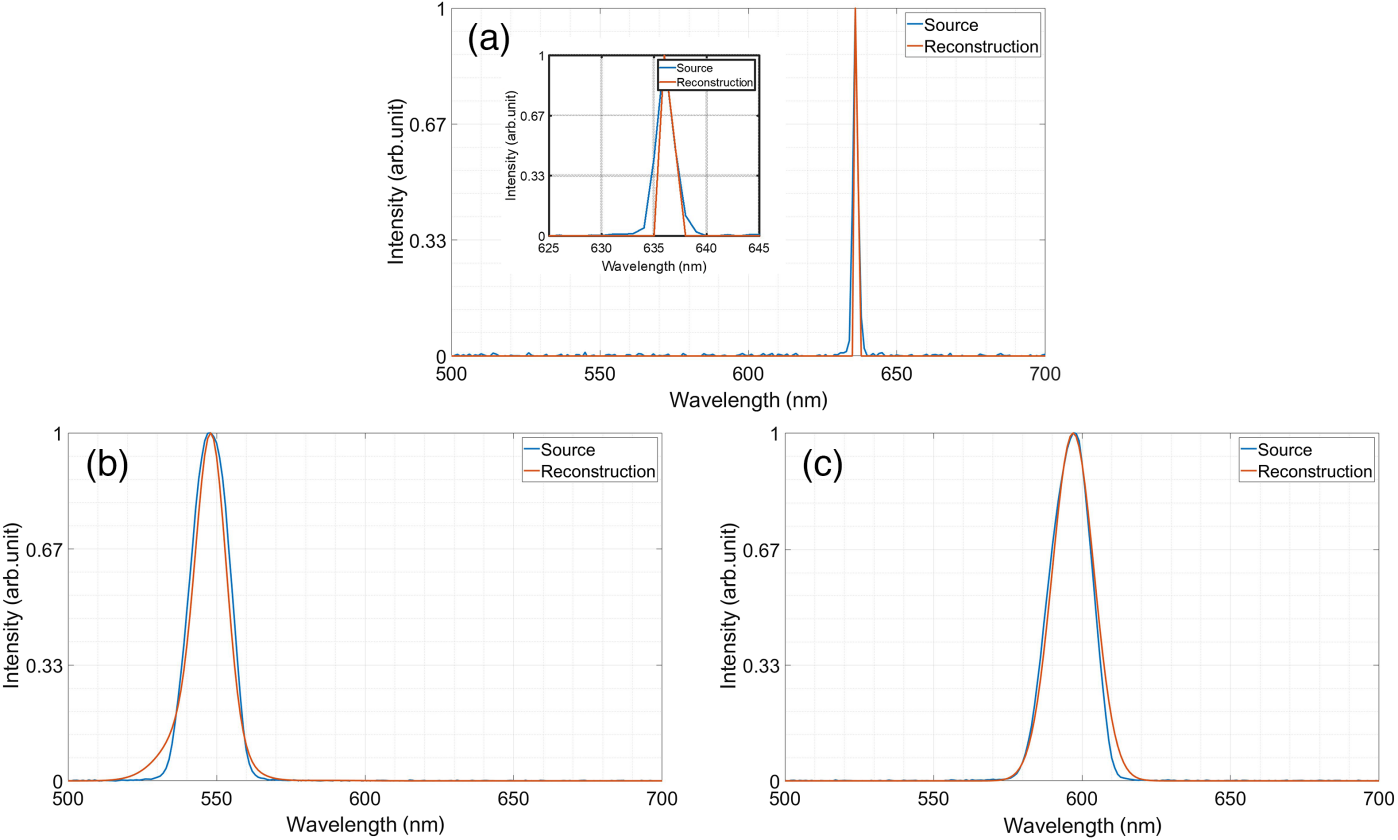
(a) Comparison of reconstructed spectra to ground truth. Spectral reconstructions of known spectral sources. Reconstruction of a narrow red filter (a) and broadband filters with a central wavelength peak at (b) 550 nm and (c) 600 nm, respectively.

To test the spectral resolution, we captured with our system the light passing spectral filters with known spectral transmission. Then we compared the reconstructed spectrum to the ground truth. First, we placed a red narrowband filter (632.8 nm CWL, 1 nm FWHM Edmund Optics) on the microscope stage and performed a compressed measurement. From 47 multiplexed captured measurements, $g\in R^{47}$, we reconstructed an HS data cube with N=200 spectral bands in the range of 500 to 700 nm. For the reconstruction, we used the TwIST solver.^29^ The choice of N=200 spectral bands implies a resolution of 1 nm, which is also the FWHM of the measured filter. It also complies with the resolution of the CS-MUSI.^21^^,^^24^ The reconstructed spectrum is shown in Fig. 8(a). As it can be seen, the spectral reconstruction exhibits a peak at 636 nm, which is the exact spectral location of the red filter peak that was measured with a commercial grating spectrometer. In addition to the narrow band filter measurement, we also performed compressed measurements of two different broadband filters with a central wavelength peak at 550 nm (Thorlabs FB550-10-Ø1″ bandpass filter, CWL=550±2  nm, and FWHM=10±2  nm) and 600 nm (Thorlabs FB600-10-Ø1″ bandpass filter, CWL=600±2  nm, and FWHM=10±2  nm). In these examples, we also used 47 multiplexed measurements to reconstruct 200 spectral bands with the help of the TwIST solver, with a learned dictionary^19^ as the sparsifying operator. In Figs. 8(b) and 8(c), we present the reconstructed spectra at arbitrary pixels in the images obtained with the different spectral filters, compared to the spectra of the filters that were measured with a standard grating-based spectrometer (FLAME-S Ocean Optics). The reconstruction peak-signal-to-noise ratio is 29.9, 29.6, and 32.2 dB for the red narrow, 550 and 600 nm filters, respectively.

### 3.3 Optical Throughput

**Fig. 9:**
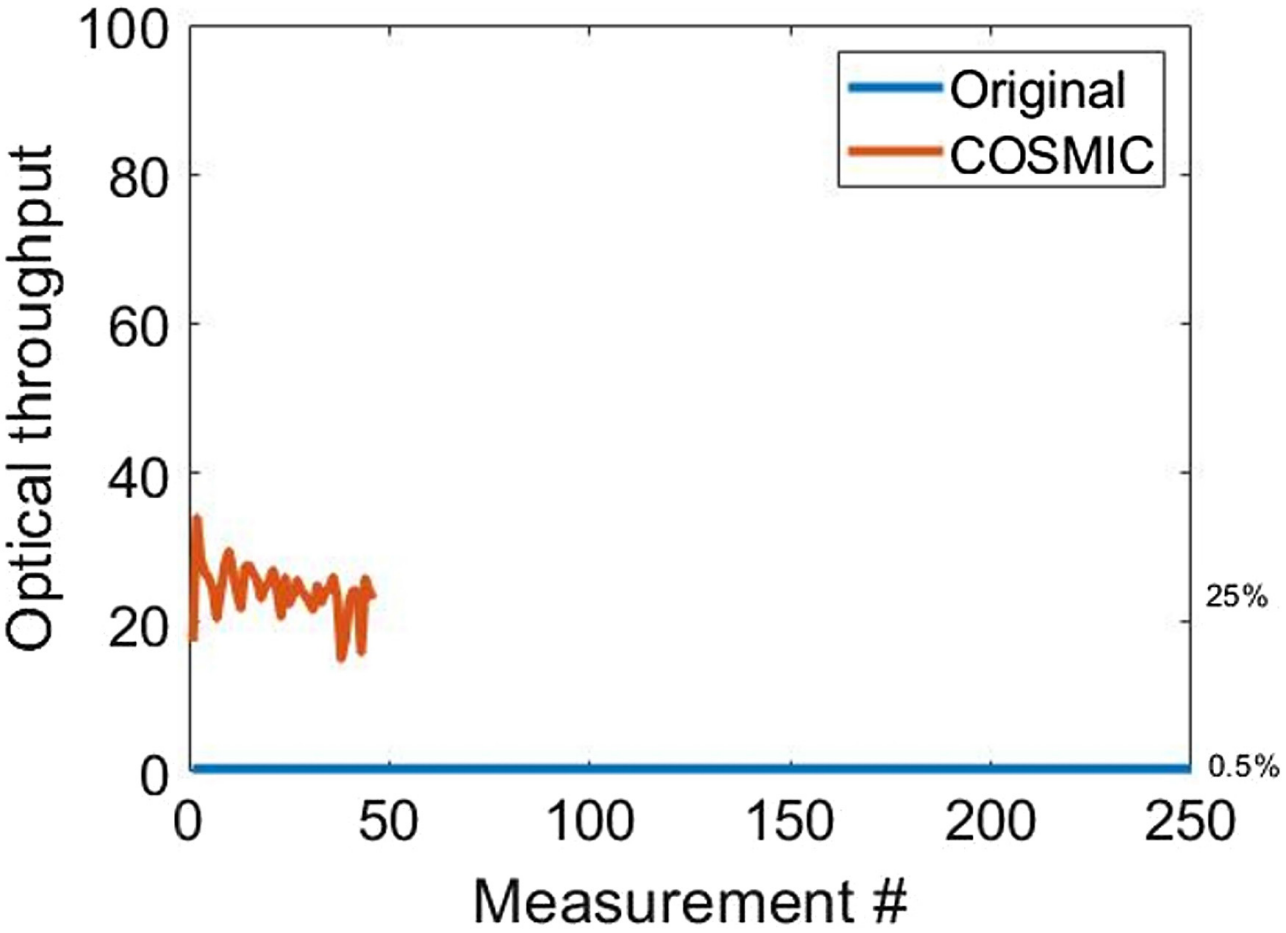
Optical throughput (∼25%) and number of measurements of our system compared to a conventional system that has an optical throughput of ∼0.5%.

An important property of our system is its high optical throughput. The high optical throughput is due to Fellgett’s multiplex advantage, initially demonstrated for Fourier transform spectrometers,^30^ but known to be generic for multiplex techniques (see for example, Refs. 31–33), such as our COHSM. Figure 9 shows the optical throughput at each exposure, calculated as the area under the measured spectral transmission graph (rows in the spectral transmission map from Fig. 6) for each exposure. The average optical throughput for capturing 250 spectral bands in our system is 25%, which is two orders of magnitudes higher than the throughput of 1/250 that a perfect 250-band filter would exhibit.

## 4 Histopathology Tests

### 4.1 Samples Preparation Descriptions

To demonstrate the validity of the optical system for pathological analysis, we used lymph node biopsies stained with Hematoxylin & Eosin (H&E). Normal and cancer cells were identified by a pathologist using a brightfield microscope and they were measured by a simple color (RGB) camera. The two types of cells were marked on the image and acted as a reference. Images from the same area were then measured with our COHSM for obtaining the spectral images.

**Fig. 10:**
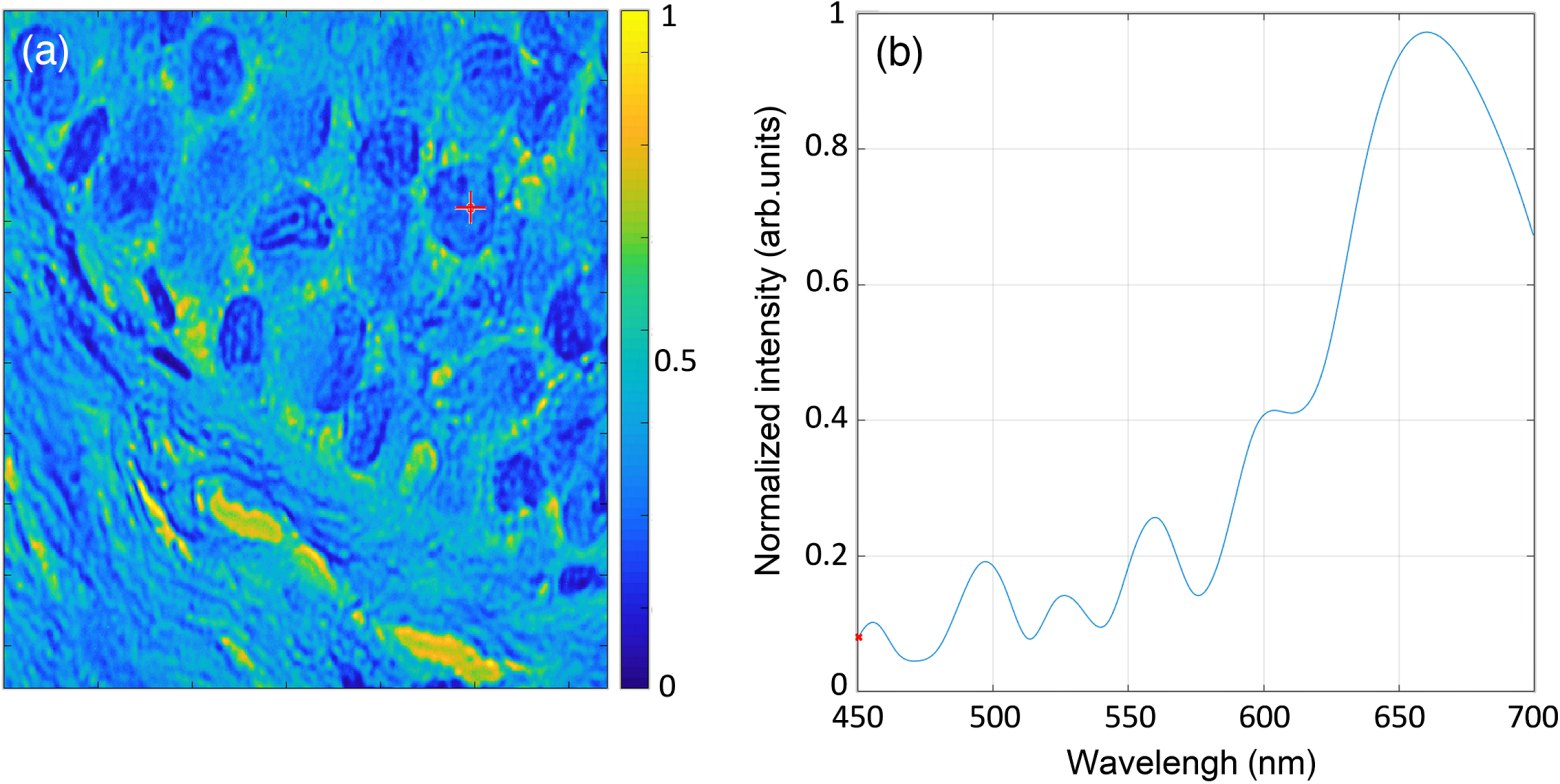
(a) Spatial marginal of a reconstructed hyperspectral image and (b) spectral distribution of the red-marked pixel in (a).

Figure 10 demonstrates an example of hyperspectral tissue data obtained with the COHSM system (Fig. 1). Figure 10(a) shows the spatial distribution obtained by integrating all spectral components for each pixel. Figure 10(b) demonstrates the reconstructed spectral distribution at the marked pixel. The hyperspectral image was reconstructed from 47 spectrally encoded images, which were captured in only 1 s. From the captured data, we reconstructed an HS datacube with 250 spectral bands in the range of 450 to 700 nm, thus yielding a compression ratio of about 5:1. The reconstruction process was done with the help of the OMP solver.^34^ The sparsifying operator that was used for reconstructing the spectral information is a learned dictionary.^19^

**Fig. 11:**
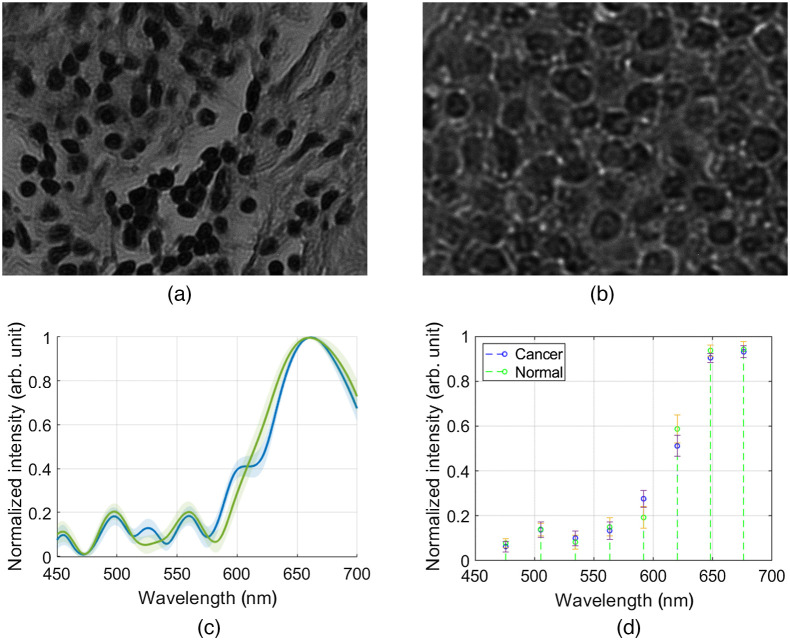
(a) Normal and (b) cancerous cells images. (c) Difference between the spectral distribution of normal and cancerous cells. The bold lines represent the mean spectra per each class, whereas the shadowed regions around the lines represent the measured variance. (d) Simulation results of the spectra in (c) as would be obtained with a multispctral imager that captures eight spectral bands.

Figure 11 compares histopathology imaging results of normal and cancerous cells. The images were taken with the COHSM system (Fig. 1) using an objective 20× and sample transmission mode.

Figures 11(a) and 11(b) show one of the captured images representing a single grayscale frame from the compressed dataset of a normal and cancerous cell’s area, respectively. Figure 11(c) shows the spectral distribution of an ensemble of healthy and cancerous cells. The average (continuous lines) and standard deviation (shaded area) of 20 healthy and cancerous pixels from Figs. 11(a) and 11(b) images are plotted. Figure 11(c) shows that the bounds of the reconstructed spectra of the healthy cells are clearly distinguished from that of cancerous cells. It is evident that it is easy to distinguish between healthy and cancerous cells visually or by means of machine learning algorithms. Machine learning algorithms can be used to classify the reconstructed spectra or applied directly to the compressed data.^8^^,^^35^ The validity of spectral imaging for various pathological and biological studies was previously demonstrated, including the identification of cell type in bladder cancer^36^ and the genetic analysis of chromosomal aberrations for both cancer and other genetic disorders identification.^37^

Figure 11(d) shows the importance of the number of spectral bands for distinguishing between normal and cancerous cells. Figure 11(d) shows the simulation of the spectra shown in Fig. 11(c) as would be obtained with a conventional multispectral imager that captures only 8 spectral bands. It can be seen that in the 8-band spectra, there is a significant overlap between the cancerous and normal spectra, which obviously may lead to high false negative rates and insufficient accuracy.

## 5 Discussion and Conclusions

We introduced COHSM—a highly efficient method for hyperspectral microscopy. COHSM spectrally encodes conventional microscopes’ illumination path to generate a set of compressive samples. By virtue of manipulating only the illumination path of the microscope, the high performance of commercial microscopes is unimpaired. The spectral encoding is realized with an LC introduced in the illumination arm and operated in a fast mode allowing full-field HS image acquisition at a rate of nearly 1 frame per s.

COSHM exhibits unique spectral imaging performance in terms of resolution, acquisition speed, and optical throughput. The method is versatile and can be easily adapted to any microscope. In the experiments, we demonstrated that the spatial resolution of our Olympus BX53 is fully maintained in the reconstructed HS images. The acquisition of HS data within ∼1  s was demonstrated, from which the HS images were reconstructed with 250 spectral bands. The system was used for histopathology testing of breast cancer biopsies showing that cancerous cells can be clearly distinguished from benign cells based on the spectral data captured with the COSHM system.

## Code, Data, and Materials Availability

The data that support the findings of this study are available from the corresponding author upon reasonable request.
